# Georgia Medical Association

**Published:** 1876-05

**Authors:** 


### Editorial.

Georgia Medical Association.—Below we present a brief outline, or synopsis, of the minutes of the Medical Association of Georgia, convened in Augusta April 19th.

The Association was called to order by the retiring President, Dr. DeSaussure Ford. After prayer by the Rev. Mr. Evans, Dr. H. F. Campbell made an address of welcome, responded to by Dr. J. M. Johnson, of Atlanta.

President J. G. Thomas was introduced by Dr. Ford, and after taking the chair, proceeded with the President's address. The doctor selected as his subject an exposition of the objects and benefits conferred by the State Board of Health, rather than a more formal written address. He exhibited thorough acquaintance with, and deep interest in, the workings of the Board.

Dr. Eugene Foster read a paper on The Effectual Means of Preventing Small-pox, including specially a more thorough Vaccination, Re-vaccination, Isolation, and Disinfection.

Dr. T. S. Hopkins, through the Secretary, a paper on Ovarian Tumor, treated by Chlorate of Potassa.

The report on Gynæcology, 1st District, read by Dr. K. P. Moore.

Dr. W. O'Daniel, Synopsis of a paper on Gynæcology.

Dr. R. M. Smith, A case of Extra Uterine Pregnancy, exhibiting the pathological specimen.

Dr. L. A. Dugas, a paper on the Pathological Peculiarities of the Negro Race.

Dr. A. W. Calhoun, a report of sixty-five cases of Operation for Cataract.

Dr. W. F. Westmoreland, a paper on Fibro Cystic Tumor of the Jaw, exhibiting several specimens.

Dr. J. C. LeHardy, a paper on the Relation of Disease and Mortality to Terrestrial and Aeral Influences.

Dr. W. H. Doughty, On the true Method of Treating Dislocation of the Scapular End of the Clavicle.

Dr. H. F. Campbell, A Report of a number of cases of

Stone in the Bladder, with considerations relating to the Etiology and Rate of Deposit of Calculous Matter in the Bladder.

Dr. A. F. Barnard, Treatment of Opium Poisoning by the Hypodermic Use of Atropia.

A communication from S. D. Gross, requesting the appointment of delegates to the American Medical Association.

Dr. J. G. Westmoreland, On the Comparative Value of Nitrate of Silver, Carbolic Acid, Iodine, and Muriated Tincture of Iron in the Treatment of Chronic Endometritis.

Dr. Robert Battey, a Club-Foot Apparatus, with some remarks upon its Use. A discussion by the body.

Dr. J. M. Johnson (by request of Dr. E. J. Kirkscey), a resolution to appoint a committee, to report at next meeting, On the Rise and Progress of Medicine in Georgia for the past Hundred Years. Committee, E. J. Kirkscey, J. M. Johnson, T. J. Word, G. E. Sussdorf, G. J. Grimes.

Dr. DeSaussure Ford, chairman of Section on Surgery for 8th District, reported cases as follows: Laceration (double lateral) of Cervix—Emmett's operation modified; Two cases of Extraction of Cataract by Graefe's method—successful; Three cases of Extirpation of Tumor without return—in one case Esmarch's bandage used—presented specimens; two cases of Lithotomy with Double Lithotome Cache—one a child five years old; three cases of Hare-Lip in the Same Family; one case of Fracture of Clavicle, treated with Sayre's Adhesive Plaster Apparatus—no deformity.

The President announced Nominating Committee: Drs. L. D. Ford, W. O'Daniel, R. Battey, R. J. Nunn, R. H. Smith, A. W. Calhoun, W. C. Musgrove, G. E. Sussdorf, T. H. Kenan, K. P. Moore, E. W. Alfriend, C. S. Strother, J. B. Roberts, E. F. Knott, and H. L. Burt.

Delegates to the American Medical Association (to convene in Philadelphia June 6th, 1876): Drs. J. P. Logan, T. S. Hopkins, G. M. McDowell, J. G. Westmoreland, L. A. Dugas, J. T. Johnson, H. F. Scott, K. P. Moore, J. C. LeHardy, DeSaussure Ford, Juriah Harriss, R. J. Nunn, R. H. Lewis, G. F. Cooper, B. M. Cromwell, W. A. Greene, G. E. Sussdorf, E. L. Crump, W. S. Kendrick, R. M. Smith, T. J. Word, R. C. Eve, W. F. Holt, J. S. Coleman, Eugene Foster, E. W. Alfriend, A. W. Calhoun, S. G. White, E. J. Kirkscey, R. F. Wright,

Sterling Eve, L. D. Ford, J. T. Banks, V. H. Taliaferro, W. A. Love, R. B. Doster.

The President appointed, on motion, delegates to the International Medical Congress, to meet in Philadelphia September 4th: Drs. J. A. Eve, Robert Battey, R. J. Nunn, W. H. Doughty, W. F. Westmoreland, D. W. Hammond, H. F. Campbell, F. A. Stanford, W. O'Daniel and J. M. Johnson.

Dr. DeSaussure Ford invited the Association to partake of the hospitalities of his home on Friday at one o'clock.

Amendments to the by-laws were adopted fixing the initiation fee at five dollars; the annual assessment at five dollars—failure to pay for two consecutive years forfeiting membership; also, preventing the publication of any of the papers presented, before publication in the volume of Transactions.

Dr. Lewis read a "Report of Cases."

By request of Dr. Foster the committee on an Inebriate Asylum was continued: Drs. Foster, Nottingham and Myers.

Dr. Foster, of the committee on criminal abortion, reported the passage of the law by the Legislature. Committee discharged.

Dr. G. M. McDowell made some remarks upon the influence of East winds, suggested by the paper of Dr. LeHardy.

The Nominating Committee made a partial report, which was confirmed by the Association:

For President—Dr. Robert Battey.

For First Vice-President—Dr. K. P. Moore.

For Second Vice-President—Dr. A. W. Calhoun.

For Censor—Dr. R. M. Smith.

(Secretary and Treasurer are continued in office by the Constitution.)

A session was held Thursday night for the Annual Address, Dr. A. S. Campbell Orator. A fine audience was present, and were treated to an elegant oration.

Macon was selected as the place for the next annual meeting.

The Committee on Necrology reported a sketch of the life of Drs. C. B. Nottingham, of Macon, and W. G. McBride, of Washington county, and introduced resolutions expressing the regret of the Association. Eulogies were pronounced by by Drs. O'Daniel, Sussdorf, Thomas and Battey.

A resolution was introduced by Dr. Alfriend, endorsing the action of the Legislature in establishing a State Board of Health; appointing a committee to memorialize the Legislature to vote a sufficient appropriation to sustain it properly; and calling on the County Boards of Health to arouse the citizens to an appreciation of the benefits to be derived from a State Board of Health. (Committee to be appointed at the President's leisure.)

A committee of three was appointed to memorialize the Legislature for the enactment of laws for compulsory vaccination and re-vaccination. Committee: Drs. Foster, Logan and W. F. Westmoreland.

Dr. L. A. Dugas introduced a resolution amending the by-laws so that all papers should be placed in the hands of the Secretary within two weeks after adjournment. Amendment adopted.

Dr. H. F. Campbell presented the title of a paper to be forwarded to the Secretary: The Ligation of Arteries as a means of Curing Inflammation and Preventing Gangrene, and of Arresting its Progress in Wounded and Inflamed Limbs.

Dr. Hiram Smith reported a case of Unusual Fracture of the Fibula.

The Nominating Committee completed their report, as follows:

Committee of Arrangements—Drs. Sussdorf, Hall, Burgess, Holt, and Wright.

Commiitee on Publication—Drs. J. T. Johnson, W. O'Daniel, J. B. Baird, W. S. Armstrong and W. S. Kendrick.

Committee on Necrology—Drs. J. M. Johnson, J. C. Blackshear, J. C. LeHardy, J. C. Bacon, and R. C. Eve.

*Congressional Sections*—First District—Practice of Medicine—Drs. J. G. Thomas, E. L. Crump, B. S. Purse. Surgery—T. J. Charlton, W. G. Bullock, R. P. Myers. Gynæcology—J. C. LeHardy, T. Smith, L. B. Bouchelle.

Second District—Practice of Medicine—P. L. Hillsman, R. B. Doster, W. M. Bruce. Surgery—R. T. Kendrick, W. J. Harrell, S. W. Burney. Gynæcology—J. A. Butts, D. S. Brandon, E. W. Alfriend.

Third District—Practice of Medicine—S. B. Hawkins, J. H. Stephens, J. W. Tucker. Surgery—H. V. Johnson, T. F.

Walker, J. B. Hinkle. Gynæcology—W. J. Reese, Y. H. Morgan, G. F. Cooper.

Fourth District—Practice of Medicine—T. F. Brewster, E. L. Bardwell, C. B. Leitner. Surgery—A. B. Calhoun, Geo. Grimes, F. L. Wisdom. Gynæcology—F. A. Stanford, J. E. Bacon, T. J. Word.

Fifth District—Practice of Medicine—J. T. Banks, R. B. Murchison, John G. Westmoreland. Surgery—W. F. Westmoreland, C. S. Strother, A. W. Calhoun. Gynæcology—T. S. Powell, Paul Faver, R. C. Word.

Sixth District—Practice of Medicine—W. R. Burgess, C. H. Hall, W. A. Greene. Surgery—D. W. Hammond, W. O'Daniel, G. E. Sussdorf. Gynæcology—T. H. Kenan, A. Kingman, J. E. Blackshear.

Seventh District—Practice of Medicine—J. B. Underwood, W. D. Hoyt, William Farrell. Surgery—C. P. Gordon, J. B. S. Holmes, M. W. Gray. Gynæcology—Robert Battey, W. P. Harden, J. M. Gregory.

Eighth District—Practice of Medicine—E. H. Hunter, Eugene Foster, W. W. Battey. Surgery—L. A. Dugas, DeSaussure Ford, A. S. Campbell. Gynæcology—J. A. Eve, J. S. Coleman, H. F. Campbell.

Ninth District—Practice of Medicine—W. T. Hollingsworth, C. W. Long, J. E. Pope. Surgery—A. J. Shaffer, J. W. Bailey, R. M. Smith. Gynæcology—J. B. Carlton, J. S. Simmons, H. R. J. Long.

The Board of Censors made a report disclaiming any intention of reflecting on any member of the Middle Georgia Medical Society in their report of last year.

Dr. Sussdorf introduced a resolution of thanks for the generous hospitality of the profession and citizens of Augusta. This was enthusiastically adopted. After a brief address of thanks by the President, the Association adjourned, to meet in Macon on the third Wednesday in April, 1877.

(It would be unjust to the sense of gratitude of the visiting members to fail to mention the hospitality extended individually by the Physicians of Augusta, as well as the banquet of Thursday night, and the delightful trip up the canal after adjournment on Friday. It will be long ere the Association forgets such consideration and attention.)

